# A TLR7 agonist strengthens T and NK cell function during BRAF‐targeted therapy in a preclinical melanoma model

**DOI:** 10.1002/ijc.32777

**Published:** 2019-12-04

**Authors:** Lydia Bellmann, Giuseppe Cappellano, Johanna F. Schachtl‐Riess, Anastasia Prokopi, Athanasios Seretis, Daniela Ortner, Christoph H. Tripp, Constance E. Brinckerhoff, David W. Mullins, Patrizia Stoitzner

**Affiliations:** ^1^ Department of Dermatology, Venereology & Allergology Medical University of Innsbruck Innsbruck Austria; ^2^ Department of Medicine and Biochemistry, Geisel School of Medicine at Dartmouth Norris Cotton Cancer Center Lebanon NH; ^3^ Microbiology and Immunology, Geisel School of Medicine at Dartmouth Norris Cotton Cancer Center Lebanon NH

**Keywords:** BRAF inhibitor resistance, melanoma, targeted therapy, T cell and NK cell immunity

## Abstract

Therapeutic success of targeted therapy with BRAF inhibitors (BRAFi) for melanoma is limited by resistance development. Observations from preclinical mouse models and recent insights into the immunological effects caused by BRAFi give promise for future development of combination therapy for human melanoma. In our study, we used the transplantable D4M melanoma mouse model with the BRAF^V600E^ mutation and concomitant PTEN loss in order to characterize alterations in tumor‐infiltrating effector immune cells when tumors become resistant to BRAFi. We found that BRAFi‐sensitive tumors displayed a pronounced inflammatory milieu characterized by high levels of cytokines and chemokines accompanied by an infiltration of T and NK cells. The tumor‐infiltrating effector cells were activated and produced high levels of IFN‐γ, TNF‐α and granzyme B. When tumors became resistant and progressively grew, they reverted to a low immunogenic state similar to untreated tumors as reflected by low mRNA levels of proinflammatory cytokines and chemokines and fewer tumor‐infiltrating T and NK cells. Moreover, these T and NK cells were functionally impaired in comparison to their counterparts in BRAFi‐sensitive tumors. Their effector cell function could be restored by additional peritumoral treatment with the TLR7 agonist imiquimod, a clinically approved agent for nonmelanoma skin cancer. Indeed, resistance to BRAFi therapy was delayed and accompanied by high numbers of activated T and NK cells in tumors. Thus, combining BRAFi with an immune stimulating agent such as a TLR ligand could be a promising alternative approach for the treatment of melanoma.

## Abstract 2

**What's new?**

While inhibitors targeting mutant BRAF proteins can induce melanoma regression, many tumors become resistant to these agents, possibly owing to immunological effects of BRAF inhibitor therapy. Here, using a preclinical mouse model, the authors show that during the early treatment phase with BRAF inhibitors, melanomas are highly immunogenic, with infiltrating T cells and natural killer cells. When resistance develops, however, tumors regress toward low immunogenicity, similar to untreated tumors. Experiments show that in the BRAF‐sensitive phase, peritumoral injection of the TLR7 ligand imiquimod preserves immunogenicity and delays resistance, thus representing a potentially effective novel therapeutic strategy for melanoma.

**Abbreviations**
BRAFi: BRAF inhibitor
IFN: interferon
mAb: monoclonal antibody
MAPK: mitogen‐activated protein kinase
MDSC: myeloid‐derived suppressor cell
MEKi: MEK inhibitor
NK cell: Natural killer cell
PD‐1: Programmed cell death protein 1
PD‐L: Programmed cell death 1 ligand
PI3K: phosphoinositide 3‐kinase
RT‐qPCR: real‐time quantitative PCR
s.c.: subcutaneous
Tbp: TATA binding protein
TIM‐3: T‐cell immunoglobulin and mucin domain 3
TLR: toll‐like receptor
TNF: tumor necrosis factor
Treg: regulatory T cell

## Introduction

Melanoma of the skin belongs to the 10 most common cancer types both in the US and Europe and its incidence is rapidly increasing.^1^ It has a high mutational load with driver mutations occurring in genes regulating important signaling pathways involved in proliferation and growth such as in the mitogen‐activated protein kinase (MAPK) pathway (e.g., *BRAF* and *NRAS*), or the phosphoinositide 3‐kinase (PI3K) pathway (e.g., *PTEN*).^2^ In 60% of melanoma patients, mutations occur in the *BRAF* gene leading to an amino acid substitution of valine to glutamic acid in position 600 (BRAF^V600E^), which activates the MAPK pathway.^3^ This mutation is of clinical interest because it can be targeted with selective BRAF inhibitors (BRAFi) that have been approved for clinical use.^4^, ^5^ While BRAFi induce impressive melanoma regression, resistance to BRAFi occurs within the first year of treatment due to manifold resistance mechanisms.^6^, ^7^

BRAF inhibition causes tumor shrinkage and senescence‐like features in BRAF^V600E^ melanoma and most importantly, reverts the immunosuppressive milieu to a proinflammatory microenvironment.^8^, ^9^, ^10^ In preclinical mouse models, BRAFi treatment enhanced antitumor immunity by the recruitment of intratumoral T and NK cells and the reduction of regulatory T cells (Tregs) and myeloid‐derived suppressor cells (MDSCs).^11^, ^12^, ^13^, ^14^ In melanoma biopsies, increased expression of melanocyte differentiation antigens, that is, trp‐2, MART‐1 and gp100 was induced by BRAFi and accompanied by an infiltration of CD8^+^ T cells and a decrease in MDSCs.^15^, ^16^, ^17^, ^18^ The immunogenic effect of BRAFi is transient as indicated by a loss of tumor‐infiltrating T cells during progression.^16^, ^19^ Due to the immunological effects reported, preclinical studies tested combinations of BRAFi and/or MEK inhibitor (MEKi) with anti‐PD‐1 checkpoint blocking antibody and observed increased ratio of CD8^+^ effector T cells to Tregs in tumor biopsies.^20^, ^21^ Recently, performed clinical trials with the triple combination of BRAFi, MEKi and checkpoint inhibitor demonstrated promising response rates in subgroups of melanoma patients, but also reported high toxicities.^22^, ^23^, ^24^ A deeper understanding of the tumor microenvironmental changes during targeted therapy and how the immune system can be manipulated to potentiate responses is crucial for the development of urgently needed, alternative combinations.

Thus, we investigated the immunological alterations in BRAFi‐resistant tumors in a preclinical model of melanoma, namely, the transplantable mouse model D4M (carrying the BRAF^V600E^ mutation and PTEN loss^25^). We here demonstrate that BRAFi‐sensitive tumors showed a pronounced inflammatory milieu with an increase of activated, cytokine‐producing effector cells, whereas BRAFi‐resistant tumors displayed lower numbers of activated effector cells and resembled immunologically inert untreated tumors. We hypothesized that a TLR ligand‐mediated immune stimulation would be able to prevent this loss of immunogenicity. Recently, a study described that a novel TLR7 agonist reverted the suppressive tumor milieu leading to tumor cell killing by NK cells as well as T cells.^26^, ^27^ Moreover, topical application of imiquimod (the only TLR7 agonist approved by FDA) is used for treatment of nonmelanoma skin cancer and provide beneficial effects in melanoma patients.^28^, ^29^, ^30^ Indeed, we observed that additional treatment with imiquimod effectively delayed resistance development by shaping the effector T and NK cell immune landscape during BRAF‐targeted therapy. Our findings on tumor microenvironmental changes during BRAFi‐treatment could have implications for future therapies.

## Materials and Methods

### Mice

Breeding pairs for C57BL/6N mice were purchased from Charles River Laboratories (Sulzfeld, Germany). Experimental mice were bred and housed in the institutional animal facility at Medical University of Innsbruck. Female C56BL/6 N mice were used for experiments throughout the study. All animal experimental protocols were approved by the Austrian Federal Ministry of Science and Research (66.011/0122‐V/3b/2018) and performed according to institutional guidelines.

### Transplantable melanoma mouse model

The BRAF^V600E^/PTEN D4M‐3A (RRID:CVCL_0P27)^25^ murine melanoma cell line was kindly provided by Constance E. Brinckerhoff (Geisel School of Medicine at Dartmouth, Norris Cotton Cancer Center, Lebanon, NH). Cells were cultured in DMEM (containing high glucose and l‐glutamine #D6429, Sigma‐Aldrich, St. Louis, MO) supplemented with 5% heat‐inactivated fetal calf serum (FCS; PAN‐Biotech, Aidenbach, Germany), 50 U/ml penicillin and 50 μg/ml streptomycin (Thermo Fisher Scientific, Waltham, MA) and confirmed to be mycoplasma‐free by Venor GeM Classics Mycoplasma PCR Detection Kit (BioProducts, Stockerau, Austria). A total of 3 × 10^5^ D4M cells in phosphate buffered saline (PBS; Thermo Fisher Scientific) were subcutaneously (s.c.) injected into the flank. When a tumor was palpable, tumor growth was monitored using a digital caliper by measuring the shortest and the longest diameter of the tumor. The tumor size was calculated according to the formula: shortest diameter × longest diameter.

### *In vivo* treatment with BRAFi

When tumors reached a size of 25–35 mm^2^ (approximately after 8 days), diet was switched either to a BRAFi‐containing chow (417 mg PLX4720/kg; Plexxikon, Berkeley, CA; under a material transfer agreement) formulated into rodent diet by Research Diets, Inc. (New Brunswick, NJ) or control chow. Tumors were referred to as “BRAFi‐sensitive” when they had regressed after a week of BRAFi treatment (analyzed on Day 15–17 after transplantation). When mice were kept on the BRAFi‐containing diet, tumors started to regrow after 3–5 weeks and they were referred to as “BRAFi‐resistant” (analyzed on Day 33–65 after transplantation). Control chow‐treated mice were referred to as “untreated” (analyzed on Day 15–21 after transplantation). Untreated and BRAFi‐resistant tumor mice were sacrificed when tumors reached a size of approximately 75 mm^2^.

### *In vivo* treatment with a TLR7 agonist

TLR7 agonist (Imiquimod, InvivoGen, San Diego, CA) in PBS was injected s.c. around the tumors (peritumoral) twice per week starting at Day 19 at a dose of 2 mg/kg body weight. As control, mice were injected with the vehicle (PBS).

### Cell preparation

Tumor infiltrates were analyzed at different time points as indicated in the figure legends. For this purpose, tumors were weighed, minced, and digested with 250 μg/ml Collagenase D (Roche, Basel, Switzerland) and 300 μg/ml DNase I (Roche) for 45 min at 37°C. Tumor pieces were passed through a 40 μm cell strainer (Corning, NY) to obtain single‐cell suspensions for use in flow cytometry.

### Flow cytometry

Dead cells were excluded using the fixable viability dye eFluor780 (Thermo Fisher Scientific). Nonspecific FcR‐mediated antibody staining was blocked by incubation for 15 min with antimouse CD16/CD32 mAb (clone: 2.4G2, TONBO Biosciences, San Diego, CA). Stainings with fluorophore‐labeled mAbs (purchased from BD Biosciences, Franklin Lakes, NJ or BioLegend, San Diego, CA) for CD3 (clone 17A2), CD4 (clone GK1.5), CD8a (clone 53–6.7), CD11b (clone M1/70), CD45 (clone 30‐F11), CD69 (clone 1.2F3), MHC class I/H2‐k(b) (clone AF6‐88.5), NK1.1 (clone PK136), NKG2D (clone CX5), PD‐1 (clone RMP1‐30) and TIM‐3 (clone RMT3‐23) were performed for 15 min at 4°C. FMO or isotype‐matched antibodies were used as controls. Immune cells were always pregated on single, viable CD45^+^ cells. To analyze TNF‐α and IFN‐γ production, up to 1.5 × 10^6^ cells were restimulated with PMA (50 ng/ml, Sigma‐Aldrich) and ionomycin (1 μg/ml, Sigma‐Aldrich) for 4 hr in the presence of Brefeldin A (Thermo Fisher Scientific). To analyze granzyme B production, up to 1.5 × 10^6^ cells were restimulated with PMA (50 ng/ml), ionomycin (1 μg/ml) and IL‐15 (100 ng/ml, PeproTech, Rocky Hill, NJ) for 14 hr, in the last 3 hr in the presence of Brefeldin A. For intracellular staining, fixation and permeabilization were done according to the manufacturer's protocol (BD Biosciences, Cytofix/Cytoperm kit) and cells were incubated with fluorophore‐labeled mAbs for granzyme B (clone NGZB), TNF‐α (clone MP6‐XT22) and IFN‐γ (clone XMG1.2). Samples were measured using a FACS Canto II (BD Biosciences) and analyzed using FlowJo software (BD Biosciences).

### Real‐time quantitative PCR

Total RNA was extracted from tumor tissue by using Trizol Reagent (Thermo Fisher Scientific) according to the manufacturer's protocol. RNA quality was confirmed by electrophoresis on a 1% agarose gel in 2× RNA loading dye (Thermo Fisher Scientific). For genomic DNA removal, the RapidOut DNA Removal Kit (Thermo Fisher Scientific) was used. For cDNA synthesis, the SuperScript II Reverse Transcription Kit (Thermo Fisher Scientific) with random hexamers (Microsynth, Balgach, Switzerland) was employed. Real‐time quantitative polymerase chain reaction (RT‐qPCR) analysis was performed on a Bio‐Rad CFX96 (Hercules, CA) using the Brilliant III Ultra‐Fast Quantitative PCR Kit (Agilent technologies, Santa Clara, CA) and TaqMan probes. Probes and primers were purchased from Thermo Fisher Scientific (CCL2 Mm00441242_m1, CCL3 Mm00441259_g1, CCL4 Mm00443111_m1, CXCL9 Mm00434946_m1, CXCL10 Mm00445235_m1, Galectin‐9 (Lgals9) Mm00495295_m1, Gp100 Mm00498996_m1, H60b Mm04243254_m1, H60c Mm04243526_m1, IL‐10 Mm00439614_m1, IL‐12a Mm00434165_m1, IL‐12b Mm01288989_m1, IL‐15 Mm00434210_m1, IL‐18 Mm00434225_m1, PD‐L1 Mm00452054_m1, PD‐L2 Mm00451734_m1, Rae1 Mm00558293_g1, TGF‐β1 Mm01178820_m1, Trp‐2 Mm01225584_m1, Ulbp1 Mm01180648_m1). Sequences for probes and primers specific for TATA binding protein (Tbp) were synthesized by Microsynth.

### Statistical analysis

Data are presented as mean ± SD. Statistical analysis was performed using GraphPad Prism 8.0 (GraphPad Software, San Diego, CA). After examining for normality using D'Agostino and Pearson normality test, either two‐tailed unpaired Student's *t*‐test (parametric) or Mann–Whitney test (nonparametric) was used to determine the statistical significance between two groups. For more groups, one‐way ANOVA followed by Tukey's multiple comparison test (parametric) or Kruskal–Wallis test followed by Dunn's multiple comparison test (nonparametric) was used. The exact numbers of tumors used per experiment are indicated in the corresponding figure legends. A value of *p* ≤ 0.05 was considered statistically significant (*), ≤0.01 very significant (**), ≤0.001 highly significant (***) and ≤0.0001 extremely significant (****).

## Results

### BRAFi treatment causes a transient infiltration of T and NK cells

**Figure 1:**
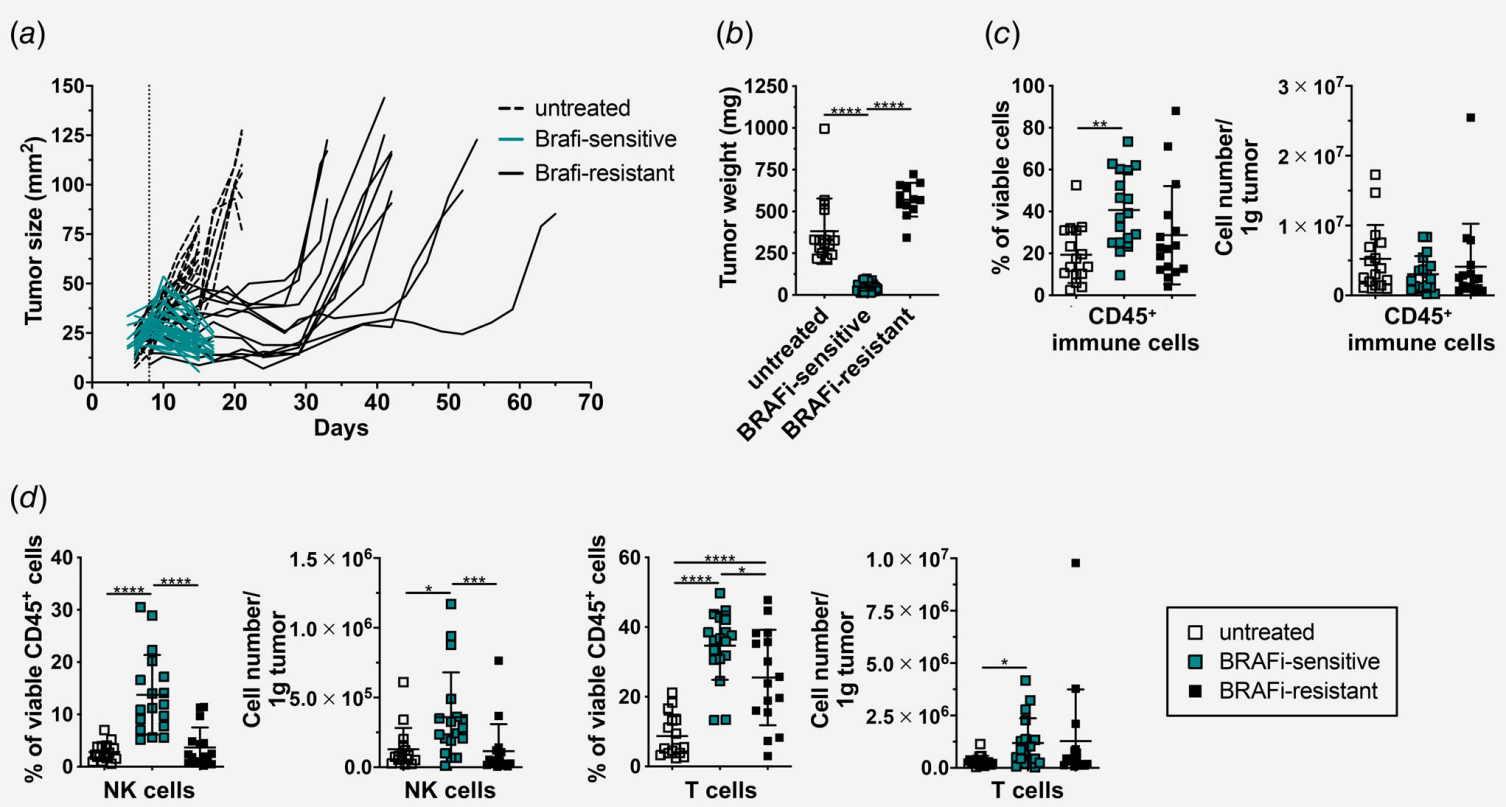
BRAFi treatment causes a transient infiltration of T and NK cells. (*a*) Tumor growth curve upon s.c. injection with 3 × 10^5^ D4M cells. On Day 8 (dotted vertical line), mice received either control chow (dashed lines = untreated) or BRAFi‐containing chow (blue and black lines). One representative graph is shown (*n* ≥ 12/group). (*b*–*d*) Tumors from mice shown in (*a*) were analyzed, and (*b*) tumor weight was measured. (*c*) Flow cytometry analysis of tumor‐infiltrating CD45^+^ immune cells, (*d*) tumor‐infiltrating NK cells (NK1.1^+^) and T cells (CD3^+^) from untreated, BRAFi‐sensitive and BRAFi‐resistant tumors. Percentages and cell numbers/g tumor are shown as summary graphs for at least two independent experiments (*n* ≥ 16/group). [Color figure can be viewed at http://wileyonlinelibrary.com]

For our study, we used the recently described BRAFV^600E^‐mutant cell line D4M‐3A (D4M), which was generated from a Tyr::CreER;Braf^CA^;Pten^lox/lox^ transgenic melanoma mouse model and demonstrated sensitivity to BRAFi.^25^ The D4M melanoma cells were injected subcutaneously (s.c.) into the flank of C57BL/6 mice and tumors reached a size of 25–35 mm^2^ within 8 days after transplantation. At this time point, we started the treatment with BRAFi‐containing chow (blue and black lines) or control chow (dashed black lines; Fig. 1 *a*). In the control, untreated group, tumors reached their maximum size within 15–21 days after transplantation. With BRAFi‐containing chow, tumors were reduced in size within 1 week of treatment and were called BRAFi‐sensitive tumors (blue lines). Treatment with BRAFi was able to control tumor growth for 3–5 weeks before resistance developed (BRAFi‐resistant tumors, black lines). When we compared the tumor weight, we found that the tumor weights in BRAFi‐sensitive mice were significantly lower than untreated and BRAFi‐resistant mice (Fig. 1 *b*). This categorization into those three groups reflects the course of biopsy sampling under BRAFi therapy in patients. Thus, we investigated the alterations in tumor‐infiltrating immune cells by flow cytometry.

Using the gating strategy depicted in Supporting Information Figure S1*a*, we first determined the proportion of viable CD45^+^ immune cells and their numbers per gram of tumor. As shown in Figure 1 *c*, we found a significant increase in the percentages of CD45^+^ immune cells within the BRAFi‐sensitive tumors in comparison with untreated tumors, however, this was not reflected in absolute numbers per gram of tumor tissue. In BRAFi‐sensitive tumors, we detected more T and NK cells when compared to untreated tumors (Fig. 1 *d*). Interestingly, these infiltrating NK cells were decreased in the resistance phase, whereas the changes in T cell numbers were not as pronounced (Fig. 1 *d*). The percentages of CD45^+^ immune cells, T and NK cells from untreated and BRAFi‐resistant tumors were comparable to tumors analyzed on Day 8, which is the time point when BRAFi treatment was initiated (Supporting Information Fig. S1*b*).

We conclude that the transplantable D4M BRAF^V600E^ mouse melanoma model responds to BRAFi treatment with initial tumor reduction followed by resistance development, as observed in patients.^7^ BRAFi‐sensitive tumors are infiltrated by T and NK cells, however, these cells are diminished during resistance development.

### BRAFi‐sensitive and resistant tumors display distinct chemokine, cytokine and tumor antigen expression patterns

**Figure 2:**
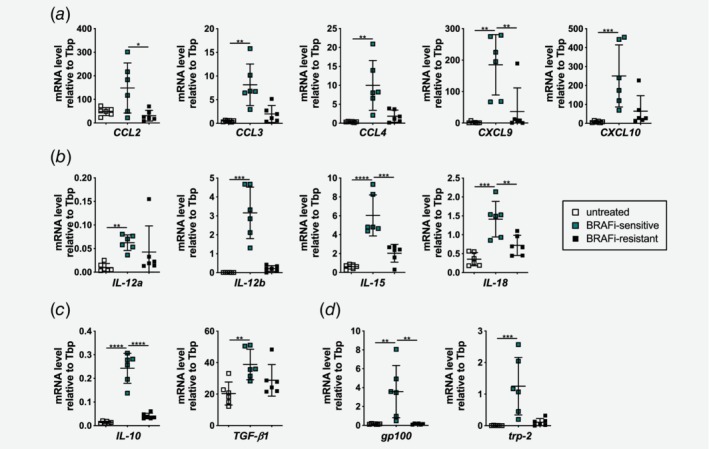
BRAFi‐sensitive and resistant tumors display distinct chemokine, cytokine and tumor antigen expression patterns. (*a*–*d*) RT‐qPCR was performed with untreated, BRAFi‐sensitive and BRAFi‐resistant tumors to determine mRNA levels for (*a*) chemokines *CCL2*, *CCL3*, *CCL4*, *CXCL9*, *CXCL10*, (*b*) proinflammatory cytokines *IL‐12a*, *IL‐12b*, *IL‐15*, *IL‐18*, (*c*) immunosuppressive cytokines *IL‐10* and *TGF‐β1* and (*d*) melanoma‐associated antigens *gp100 and trp‐2* relative to *TATA* binding protein (Tbp; *n* = 6/group). [Color figure can be viewed at http://wileyonlinelibrary.com]

To further investigate the changes in the tumor microenvironment upon BRAFi treatment, we performed RT‐qPCR on untreated, BRAFi‐sensitive and resistant tumors. First, we analyzed the expression of chemokines known to be involved in the recruitment of T and NK cells to tumors.^31^ We detected increased mRNA levels for *CCL2*, *CCL3*, *CCL4*, *CXCL9* and *CXCL10* in BRAFi‐sensitive tumors in comparison to untreated tumors. The expression of all these chemokines was downregulated upon resistance development (Fig. 2 *a*). We then screened for the expression of proinflammatory and immunosuppressive cytokines implicated in antitumor immunity.^32^ IL‐12a, IL‐12b, IL‐15 and IL‐18 are cytokines that are crucial for T and NK cell function. As shown in Figure 2 *b*, we found that these cytokines were significantly upregulated in BRAFi‐sensitive tumors when compared to untreated tumors. In line with the chemokine data, these cytokines also showed lower mRNA expression levels upon resistance development indicating a transient proinflammatory immune response in BRAFi‐sensitive tumors (Fig. 2 *b*). Interestingly, the two immunosuppressive cytokines *IL‐10* and *TGF‐β1* were also highly expressed in BRAFi‐sensitive tumors. *IL‐10* decreased in BRAFi‐resistant tumors whereas *TGF‐β1* mRNA was just marginally changed in BRAFi‐resistant tumors (Fig. 2 *c*). To identify potential antigenic targets for T‐cell recognition, we analyzed the expression of known melanoma‐associated antigens. Upon BRAFi therapy mRNA levels for *gp100* and *trp‐2* were strongly upregulated, which were subsequently lost in the resistance phase (Fig. 2 *d*).

In summary, our results show that the infiltration of immune cells is accompanied by a transient expression of chemokines in BRAFi‐treated tumors. Furthermore, those tumors show a high expression of melanoma‐associated antigens. Although BRAFi therapy *per se* leads to the induction of a proinflammatory microenvironment, immunosuppressive cytokines are also coexpressed which might be the first sign of immunosuppression developing in those tumors.

### Tumor‐infiltrating T and NK cells display an activated phenotype upon BRAFi treatment

**Figure 3:**
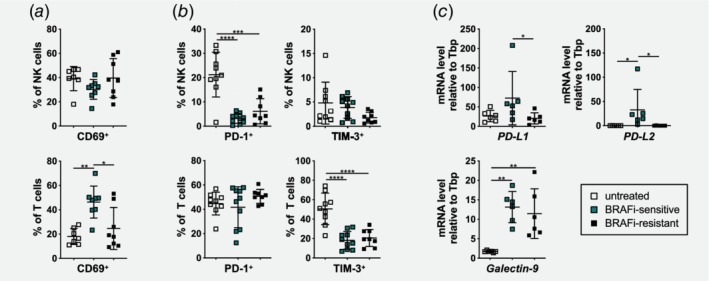
Tumor‐infiltrating NK and T cells exhibit an activated phenotype during BRAFi treatment. (*a* and *b*) Percentages of (*a*) CD69^+^, (*b*) PD‐1^+^ and TIM‐3^+^ cells of NK and T cells infiltrating untreated, BRAFi‐sensitive and BRAFi‐resistant tumors were determined by flow cytometry. Summary graphs for at least two independent experiments are shown (*n* ≥ 7/group). (*c*) RT‐qPCR was performed with untreated, BRAFi‐sensitive and BRAFi‐resistant tumors to determine mRNA levels for the inhibitory ligands *PD‐L1*, *PD‐L2* and *galectin‐9* relative to *TATA* binding protein (Tbp; *n* = 6/group). [Color figure can be viewed at http://wileyonlinelibrary.com]

In order to understand whether recruited effector cells are activated and therefore can mediate cytotoxicity in the tumor, we further investigated the phenotype of T and NK cells in D4M tumors during BRAFi treatment. Flow cytometry analysis (gating shown in Supporting Information Fig. S2*a*) of the three tumor phases revealed a strong upregulation of the early cell surface activation marker CD69 in BRAFi‐sensitive tumors on T cells, whereas NK cells displayed unchanged CD69 levels upon BRAFi therapy (Fig. 3 *a*). Furthermore, the expression of PD‐1 and TIM‐3, receptors that are implicated in immunosuppression,^33^ was studied on the infiltrating effector cells. PD‐1 was detected on 20% of NK cells in untreated tumors, but only approximately 5% of NK cells expressed PD‐1 after BRAFi treatment (Fig. 3 *b*). Interestingly, also few PD‐1^+^ NK cells were found in tumors analyzed on Day 8 after tumor transplantation (start of BRAFi treatment; Supporting Information Fig. S2*b*). TIM‐3 was only expressed on a small fraction of NK cells and this percentage did not change upon BRAFi treatment (Fig. 3 *b*, Supporting Information Fig. S2*b*). In contrast, half of the tumor‐infiltrating T cells expressed PD‐1 and TIM‐3 (Fig. 3 *b*, Supporting Information Fig. S2*b*). Interestingly, the percentage of PD‐1^+^ T cells did not change upon BRAFi treatment, whereas TIM‐3 was found on 20% of T cells after BRAFi treatment in sensitive and resistant tumors (Fig. 3 *b*). As a next step, the expression levels of the corresponding ligands for PD‐1 (PD‐L1 and PD‐L2) and for TIM‐3 (Galectin‐9) were examined by RT‐qPCR. We found that in untreated D4M tumors, these ligands were expressed at low levels (Fig. 3 *c*). On the contrary, in BRAFi‐sensitive tumors, the inhibitory ligands *PD‐L1* as well as *PD‐L2* were expressed at higher levels than in BRAFi‐resistant tumors. In addition, expression of *galectin‐9* was increased upon BRAFi treatment but there were no significant changes between sensitive and resistant phase (Fig. 3 *c*).

These results show that tumor‐infiltrating T and NK cells display an activated phenotype during BRAFi therapy with high levels of CD69, and low to moderate levels of inhibitory PD‐1 and TIM‐3. Moreover, transient upregulation of PD‐L1, PD‐L2 and galectin‐9 in the tumor microenvironment indicate evolving tumor escape mechanisms. These findings demonstrate that BRAFi‐sensitive tumors are highly immunogenic.

### T and NK cells from BRAFi‐sensitive tumors produce more cytokines and toxic granzyme B and possess the ability to detect tumor cells

**Figure 4:**
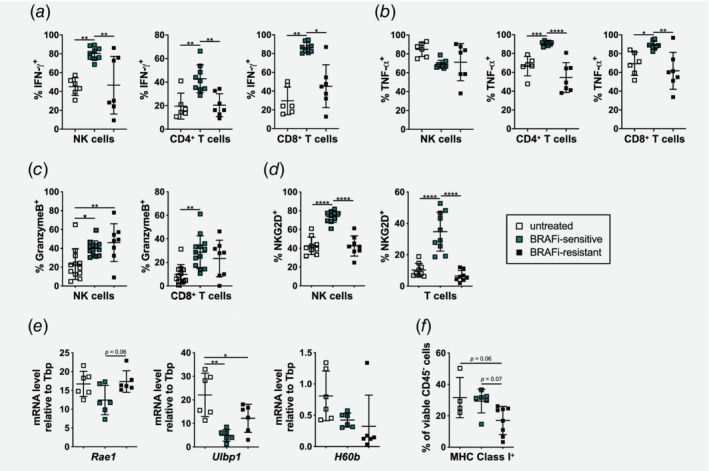
NK and T cells in BRAFi‐sensitive tumors produce cytokines and possess the ability to kill tumors cells. (*a* and *b*) Flow cytometry analysis showing the percentages of (*a*) IFN‐γ^+^ and (*b*) TNF‐α^+^ cells of CD4^+^ and CD8^+^ T cells as well as NK cells infiltrating untreated, BRAFi‐sensitive and BRAFi‐resistant tumors evaluated after *in vitro* restimulation. Summary graphs for two independent experiments are shown (*n* ≥ 6/group). (*c*) Flow cytometry analysis showing the percentages of granzyme B^+^ cells of NK and CD8^+^ T cells infiltrating untreated, BRAFi‐sensitive and BRAFi‐resistant after *in vitro* restimulation. Summary graphs for two independent experiments are shown (*n* ≥ 8/group). (*d*) Percentages of NKG2D^+^ cells of NK and T cells infiltrating untreated, BRAFi‐sensitive and BRAFi‐resistant tumors were determined by flow cytometry. Summary graphs for two independent experiments are shown (*n* ≥ 8/group). (*e*) RT‐qPCR was performed with untreated, BRAFi‐sensitive and BRAFi‐resistant tumors to determine mRNA levels for the NKGD2 ligands *Rae1*, *Ulbp1* and *H60b* relative to *TATA binding protein* (Tbp) (*n* ≥ 5/group). (*f*) Percentages of MHC‐class I^+^ cells of viable CD45^−^ tumor cells infiltrating untreated, BRAFi‐sensitive and BRAFi‐resistant tumors were determined by flow cytometry analysis. Summary graph for one experiment is shown (*n* ≥ 4/group). [Color figure can be viewed at http://wileyonlinelibrary.com]

To investigate if NK and T cells are functional in BRAFi‐sensitive tumors, we restimulated tumor cell suspensions *in vitro* with PMA and ionomycin and analyzed cytokine production by flow cytometry (gating shown in Supporting Information Fig. S3*a*). We found that NK cells, CD4^+^ T cells and CD8^+^ T cells produced more IFN‐γ in BRAFi‐sensitive tumors in comparison to untreated and BRAFi‐resistant tumors (Fig. 4 *a*). TNF‐α secretion did not change in NK cells, however, more TNF‐α^+^ cells were detected in CD4^+^ T cells and CD8^+^ T cells from BRAFi‐sensitive tumors (Fig. 4 *b*). In order to understand whether infiltrating effector cells are capable to induce apoptosis, we analyzed the intracellular granzyme B production after *in vitro* restimulation with PMA, ionomycin and IL‐15 (gating shown in Supporting Information Fig. S3*a*). NK and CD8^+^ T cells contained more granzyme B‐positive cells during BRAFi treatment, and these levels did not change during resistance development (Fig. 4 *c*).

Next, we investigated if the tumor‐infiltrating T and NK cells could maintain the capacity to recognize tumor cells during BRAFi treatment. For this purpose, we assessed the expression of the activating receptor NKG2D that can mediate cytotoxicity by NK and T cells (gating shown in Supporting Information Fig. S3*b*). The percentages of NKG2D^+^ cells were significantly higher on both NK and T cells in BRAFi‐sensitive mice compared to untreated and BRAFi‐resistant mice (Fig. 4 *d*). Tumors analyzed at Day 8 prior to BRAFi initiation showed slightly higher NKG2D expression on NK and T cells compared to untreated Day 15 tumors (Supporting Information Fig. S3*c*). Furthermore, analysis of the coexpression of the activating receptor NKG2D with the inhibitory receptors PD‐1 and TIM‐3 on NK cells revealed, that the percentage of NKG2D and PD‐1 double positive cells was low in untreated tumors, and even significantly reduced upon BRAFi therapy (Supporting Information Fig. S4*a*). TIM‐3 and NKG2D double positive NK cells were scarce in all tumor phases (Supporting Information Fig. S4*a*). For the infiltrating T cells, there was a higher percentage of NKG2D and PD‐1 double positive cells (20%) in BRAFi‐sensitive tumors compared to untreated and resistant tumors (Supporting Information Fig. S4*b*). In addition, in BRAFi‐sensitive tumors we observed a significant higher percentage of NKG2D and TIM‐3 double positive T cells (10%) compared to resistant tumors (Supporting Information Fig. S4*b*).

NK cells can sense tumor cells *via* NKG2D ligands and subsequently eliminate them.^34^ Thus, we performed RT‐qPCR to analyze the mRNA levels of the NKG2D ligands *Rae1*, *Ulbp1*, *H60b* and *H60c* in tumors from the different phases during BRAFi treatment (Fig. 4 *e*). The mRNA expression levels for all ligands were lower in BRAFi‐sensitive tumors compared to untreated tumors. Interestingly, the expression levels of *Rae1* and *Ulbp1* were almost restored during resistance development, while *H60b* mRNA was further decreased (Fig. 4 *e*). Expression of *H60c* mRNA was not detectable.

By missing‐self recognition, NK cells can also sense the lack of MHC‐class I molecules.^35^ Therefore, we investigated MHC‐class I expression on CD45^−^ tumor cells by flow cytometry. We observed a reduction of MHC‐class I expression in BRAFi‐resistant tumors compared to BRAFi‐sensitive and untreated tumors (Fig. 4 *f*), albeit not statistically significant. Interestingly, in tumors analyzed at Day 8 before BRAFi initiation even less MHC‐class I^+^ cells were found (Supporting Information Fig. S5*a*). Nevertheless, the loss of melanoma‐associated antigens gp100 and trp‐2 shown in Figure 2 *d* suggests that T‐cell recognition of tumor cells is most likely impaired.

Taken together, we found that BRAFi treatment boosts effector function of T and NK cells, especially in the BRAFi‐sensitive phase. The frequency of effector cells producing IFN‐γ and TNF‐α was reduced during development of resistance to BRAFi, however, cytotoxic ability mediated by granzyme B seems to be preserved. NK and T cells infiltrating BRAFi‐sensitive tumors also expressed higher levels of NKG2D to recognize transformed cells, but tumor cells downregulated NKG2D ligands indicating another mechanism how BRAFi‐treated tumors might evade immune recognition. Moreover, NKG2D positive T cells partly coexpressed inhibitory molecules PD‐1 and TIM‐3.

### Peritumoral application of the TLR7A prolongs tumor growth control in BRAFi‐treated D4M melanoma

**Figure 5:**
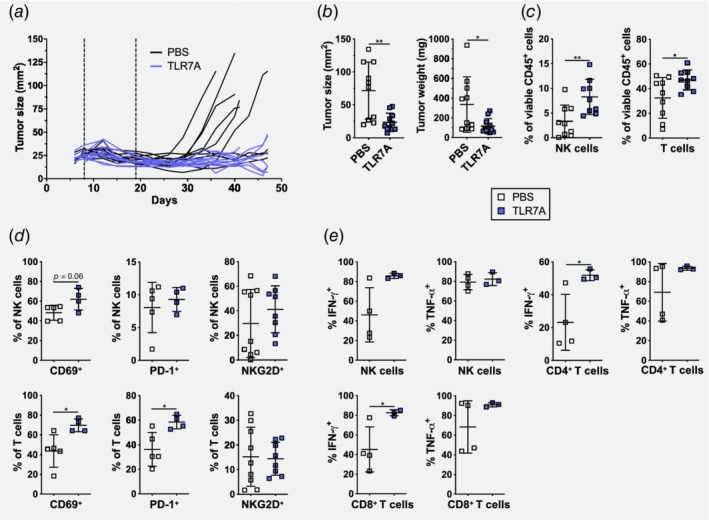
Additional application of the TLR7A during BRAFi treatment prolongs tumor growth control in D4M melanoma. (*a*) Tumor growth curve after s.c. injection with 3 × 10^5^ murine melanoma D4M cells. On Day 8 (first dotted vertical line), mice were switched to BRAFi‐containing chow and on Day 19 (second dotted vertical line) mice were given s.c. peritumorally 2 mg/kg of the TLR7A imiquimod (blue lines) twice per week or PBS (black lines). Summary graph for two independent experiments is shown (*n* = 10–11/group). (*b*) Size and weight of tumors from (*a*) were assessed at Days 41–47 or when tumors reached a size over 75 mm^2^. (*c*) Percentages of tumor‐infiltrating NK and T cells from PBS‐treated or TLR7A‐treated tumors were determined by flow cytometry. Summary graphs for two independent experiments are shown (*n* = 9/group). (*d*) Percentages of CD69^+^, PD‐1^+^ and NKG2D^+^ cells of NK and T cells infiltrating PBS‐treated or TLR7A‐treated tumors were determined by flow cytometry. Summary graphs for 1–2 experiments are shown (*n* ≥ 4/group). (*e*) Percentages of IFN‐γ^+^ and TNF‐α^+^ cells of CD4^+^ and CD8^+^ T cells as well as NK cells infiltrating PBS‐treated or TLR7A‐treated tumors were determined upon *in vitro* restimulation. Summary graph for one experiment is shown (*n* ≥ 3/group). [Color figure can be viewed at http://wileyonlinelibrary.com]

Our results so far have demonstrated that the transition from the BRAFi‐sensitive to the BRAFi‐resistant phase is accompanied by distinct changes in the immune characteristics of the tumors, with sensitive tumors being more immunogenic, whereas resistant tumors revert to an inert state. We hypothesized that immune boosting agents should resensitize the tumor microenvironment and prevent the loss of immunogenicity. Upon screening for possible TLR ligands, we selected the TLR7 agonist (TLR7A) imiquimod as it has been used in the clinics for the treatment of nonmelanoma skin cancer but also melanoma.^28^, ^29^, ^30^, ^36^ Furthermore, TLR7A has pleiotropic effects on T and NK cells and induces autophagic cell death in melanoma cells.^37^, ^38^ Moreover, a recent study showed that a novel TLR7A reversed NK cell anergy and induced antitumor CD8^+^ T cell responses.^26^, ^27^ Therefore, we assessed if treatment with a TLR7A as an immune modulator would be efficient in prolonging sensitivity to BRAFi. Mice were injected with 3 × 10^5^ D4M cells s.c. into the flank skin of C57BL/6. At Day 8, the treatment with BRAFi‐containing chow was initiated (Fig. 5 *a*). At Day 19 (middle of sensitive phase), mice were randomized in two groups and either treated with 2 mg/kg of the TLR7A imiquimod given peritumorally two times per week (blue lines) or with PBS (black lines; Fig. 5 *a*). As shown in Figure 5 *a*, 6 out of 10 mice treated with BRAFi+PBS became resistant to BRAFi in the observed time period of 47 days, however, in the mice treated with BRAFi plus TLR7A, no resistance to therapy developed. At the end point, tumor sizes and weights were significantly reduced in BRAFi+TLR7A treated mice (Fig. 5 *b*). Monotherapy with TLR7A had no direct effect on tumor size and tumors grew progressively (Supporting Information Fig. S5*b*).

To investigate the effect of the additional TLR7A treatment on the effector cells in the tumor microenvironment, we examined T and NK cells in these tumors by flow cytometry. We found a significant increase of T and NK cells in BRAFi+TLR7A treated tumors in comparison to BRAFi+PBS treated ones (Fig. 5 *c*). In regard to the activation status, more CD69^+^ NK and T cells were present in BRAFi+TLR7A treated tumors and PD‐1 was upregulated on T cells. In contrast, NKG2D was unaltered (Fig. 5 *d*). Upon *in vitro* restimulation, we observed that more NK and T cells produced IFN‐γ in BRAFi+TLR7A treated mice whereas no significant differences were found in TNF‐α production (Fig. 5 *e*).

We conclude that the TLR7A imiquimod prevented the loss of immunogenicity and effectively delayed the initiation of the BRAFi‐resistant phase by reshaping the immunological landscape toward maintenance of high numbers of activated and cytokine‐producing effector cells.

## Discussion

BRAF‐targeted therapy can only provide long‐term survival in a few patients due to resistance development.^7^ An interesting feature of BRAFi‐treated tumors is that they get infiltrated by immune cells.^16^, ^18^ How these cells are impacted by BRAFi treatment and how this shapes further antitumor immune responses remains incompletely understood.^39^ Preclinical transplantable and transgenic tumor models were used to understand the immunological effects of BRAFi and showed, similar to patients, increased immunogenicity of BRAFi‐sensitive tumors.^11^, ^12^, ^13^, ^14^, ^20^, ^21^, ^40^ However, very few of these studies investigated the immune infiltrate during resistance development.^21^, ^40^ Thus, the rationale of our study was to investigate changes in the immunological landscape during BRAFi therapy in a preclinical melanoma mouse model and to modulate antitumor immunity to delay resistance development. We demonstrate that in the early BRAFi treatment phase, tumors from the transplantable D4M model,^25^ carrying the BRAF^V600E^ mutation and PTEN loss, are highly immunogenic and infiltrated by activated effector T and NK cells. This picture changes quite markedly when tumors become resistant to BRAFi, as those tumors show an immunologically inert state similar to untreated tumors with lower numbers of infiltrating effector cells and less proinflammatory cytokines.

Antitumor immunity is mainly driven by T cells^41^ but the importance of NK cells in this process has gained interests recently.^42^, ^43^ As both immune cell types can directly kill tumor cells and produce cytokines to boost antitumor immunity,^44^ we focused our study on these effector immune cells. The recently described transplantable D4M melanoma mouse model^25^ responds well to BRAFi treatment and undergoes an approximately 3 weeks long BRAFi‐sensitive phase in which tumor growth is controlled. Thereafter, tumors progress in the BRAFi‐resistant phase. We performed flow cytometry analysis of effector immune cells during these two phases. The BRAFi‐sensitive phase was characterized by a massive infiltration of activated T and NK cells; however, this effect was only transient and resistant tumors displayed decreased immunogenicity, similar to untreated tumors. Our findings are in agreement with human clinical studies that observed an increase in CD8^+^ cytotoxic T cells early on during BRAFi therapy followed by a decrease during tumor progression.^16^, ^18^, ^19^ Thus, the D4M transplantable model is a highly suitable preclinical model to investigate immunological effects mediated by BRAFi and to test potential novel combination therapies. We confirmed the published data on the alterations in the T cell infiltrate, but also added new insights into the phenotype and functional properties of the tumor infiltrating T cells in sensitive and in the, so far less well‐described, resistant phase to BRAFi.

Moreover, we also characterized in more detail tumor infiltrating NK cells for which no clinical data are available from BRAFi‐treated patients. In our study, we observed that the NK cell infiltrate, likewise to the T cells, is lost during BRAFi‐resistance development in D4M murine melanoma. In other reports on preclinical mouse models, an increase in tumor‐infiltrating NK cells upon BRAFi treatment has been reported, however, the resistant phase was not investigated.^11^, ^13^, ^21^

In‐depth analysis of the tumor microenvironment confirmed that many proinflammatory cytokines and chemokines were strongly upregulated during the BRAFi‐sensitive phase explaining the infiltration of effector T and NK cells. Chemokines known to be involved in T and NK cell recruitment to tumors,^45^ such as *CCL2*, *CCL3*, *CCL4*, *CXCL9* and *CXCL10*, were increased in BRAFi‐sensitive tumors and downregulated in resistant tumors—mirroring the peak of effector cell infiltration. In contrast to our data, in another transplantable BRAF^V600E^‐murine melanoma model, BRAFi treatment led to a reduction of *CCL2* mRNA expression and a reduction in secretion of CCL2 from tumor cells.^11^ In line, Steinberg *et al*. observed less *CCL2* mRNA under BRAFi, which was restored in resistant tumors favoring MDSC recruitment. They also found no changes in *CCL3* and *CCL4* mRNA upon BRAFi.^40^ These differences to our data might result from the different tumor models, additional mutations in the models (PTEN loss), the different treatment regimens and time points used for analysis of tumors. CXCL9 and CXCL10 are produced primarily by monocytes, dendritic cells and cancer cells and attract CD4^+^ T cells, CD8^+^ T cells and NK cells.^45^ IFN‐γ and type I interferons secreted by other recruited immune cells induce higher expression of these chemokines resulting in a positive amplification loop.^46^ The T and NK cells infiltrating BRAFi‐sensitive tumors arrive in a highly immunogenic milieu with high levels of proinflammatory cytokines, such as *IL‐12*, *IL‐15* and *IL‐18*. Interestingly, anti‐inflammatory cytokines, namely, *IL‐10* and *TGF‐β1*, were also significantly higher in BRAFi‐sensitive tumors. This might indicate the development of escape mechanisms in response to BRAFi treatment. In line with patient samples,^18^ in our preclinical model BRAFi therapy also induced an increase in the melanoma‐associated antigens *gp100* and *trp‐2*, which decreased with resistance development.

The proinflammatory cytokines detected in BRAFi‐sensitive tumors are important to boost T and NK cell function,^41^ so we investigated if T and NK cells infiltrating BRAFi‐treated D4M tumors were activated and functional. In our study, we found that T and NK cells in BRAFi‐sensitive tumors display an activated phenotype described by the early activation marker CD69. The inhibitory molecules PD‐1 and TIM‐3 are upregulated upon T‐cell activation but sustained expression is considered a marker for T‐cell exhaustion but also marks exhausted NK cells.^33^ Indeed, these receptors were present on infiltrating T and NK cells in untreated tumors, however, BRAFi treatment resulted in a low expression of PD‐1 on NK cells and a low expression of TIM‐3 on T cells indicating that both cell types might be differentially inhibited in the tumor microenvironment. In line with previous human and mouse studies,^18^, ^20^ we also observed higher mRNA levels for *PD‐L1* and *PD‐L2* in the early phase of BRAFi treatment that were lowered upon resistance development. In addition, we observed a so far unreported high expression of the TIM‐3 ligand *galectin‐9* in BRAFi‐sensitive tumors that persisted during the resistant phase, probably indicating the beginning of immune escape mechanisms. Enhanced expression of all these ligands could also be an indication for IFN‐γ release in BRAFi‐sensitive tumors.^47^, ^48^

When we investigated the ability of the T and NK cells to produce cytokines and cytotoxic mediators, we detected very high levels of IFN‐γ, TNF‐α and granzyme B producing T and NK cells during the BRAFi‐sensitive phase. In line with our findings, others reported that CD8^+^ T cells infiltrating BRAF^V600E^‐metastatic melanoma lesions in patients produced more effector cytokines when tumor cells were pretreated with BRAFi^49^; and higher levels of granzyme B in BRAFi‐responsive metastatic melanoma samples were observed.^18^ Moreover, in a preclinical mouse model, more IFN‐γ/TNF‐α double producing T cells were present in BRAFi‐treated tumors.^20^ Another well‐defined activating receptor is NKG2D, which is expressed on both NK and T cells. Especially the killing of target cells by NK cells depends on a balance of various activating and inhibitory receptors, and the expression of the stress‐induced ligands for NKG2D by tumor cells results in their lysis.^34^ We found that activated T and NK cells in BRAFi‐sensitive tumors had a higher expression of NKG2D that was abrogated by resistance development. We also observed that T cells infiltrating BRAFi‐sensitive tumors significantly coexpressed the activating receptor NKG2D and the inhibitory molecules PD‐1 or TIM‐3. It has been demonstrated recently that BRAFi treatment can alter NKG2D ligand expression in BRAF^V600E^‐mutated melanoma cell lines.^50^ In agreement with this study, we also detected lower mRNA levels of the NKG2D ligands in BRAFi‐treated tumors which could facilitate the escape of tumor cells to NK cell‐mediated killing. Furthermore, altered NKG2D ligand expression during BRAFi therapy could also be a consequence of IFN‐γ release by activated T and NK cells.^51^, ^52^ Unchanged MHC‐class I levels in BRAFi‐treated tumors should make the cells susceptible to T‐cell mediated recognition and killing; however, expression of melanoma antigens is lowered upon prolonged BRAFi therapy as seen in our study and other mouse as well as human studies.^18^, ^20^, ^53^

Due to the low immunogenicity in BRAFi‐resistant tumors, we hypothesized that an immune stimulating agent could resensitize tumors to BRAFi. Several studies indicate that TLR7 ligands can affect tumor growth and boost antitumor immunity. For example, the topical administration of imiquimod resulted in tumor control in the B16.F10 melanoma mouse model,^54^, ^55^ whereas other studies characterized a novel TLR7 agonist that was able to revert the immunosuppressive tumor milieu by inducing effector T and NK cell responses.^26^, ^27^ Moreover, local treatment with the TLR7 agonist imiquimod leads to the production of cytokines such as IFN‐α and IL‐12 by dendritic cells, which has subsequent positive effects on innate and adaptive immune cells.^29^ In regards to clinical applications, imiquimod is used for the treatment of nonmelanoma skin cancer^36^ but has also been tested in melanoma patients,^28^, ^30^ in some even in combination with checkpoint blockade.^56^ So far combination with tumor targeted therapy, such as with BRAFi, have not been formally tested. Thus, our study demonstrates for the first time that BRAFi together with the TLR7A imiquimod could be synergistic in the treatment of melanoma, at least in this preclinical mouse model. The peritumoral administration of imiquimod preserved the immunogenicity of the tumor and prolonged tumor control by maintaining high numbers of activated T and NK cells during BRAFi therapy. These findings fit to the observations that imiquimod has manifold effects on the activation of NK and T cells.^26^, ^27^, ^37^ We cannot exclude that other cell types might also be affected, since imiquimod as an immune response modifier has pleiotropic effects on different immune and nonimmune cell types.^37^

In conclusion, our study demonstrates that tumor targeted therapy with BRAFi causes multiple immunological alterations in the tumor microenvironment. In addition, our findings add novel insights into how the immune landscape can be shaped by TLR7‐mediated immune stimulation in BRAF‐targeted therapy. These results could pave the way for the development of future therapies for melanoma.

## Supporting information


**Appendix S1** Supporting information

## Data Availability

Any data that support the findings of our study are included within the article and are available from the corresponding author upon reasonable request.
