# Supplementary material for: A TLR7 agonist strengthens T and NK cell function during BRAF‐targeted therapy in a preclinical melanoma model
Source: Int J Cancer. 2019 Dec 4;146(5):1409–20. doi: 10.1002/ijc.32777 (PMC7003881; doi:10.1002/ijc.32777)
Supplement: Supplementary file 1 — Appendix S1 Supporting information [file IJC-146-1409-s001.pdf]

A TLR7 agonist strengthens T and NK cell function during BRAF-targeted therapy in a preclinical melanoma model

Lydia Bellmann*, Giuseppe Cappellano*, Johanna F. Schachtl-Riess, Anastasia Prokopi, Athanasios Seretis, Daniela Ortner, Christoph H. Tripp, Constance E. Brinckerhoff, David W. Mullins, Patrizia Stoitzner

Supplementary Material

Supplementary Figure 1

Supplementary Figure 2

Supplementary Figure 3

Supplementary Figure 4

Supplementary Figure 5

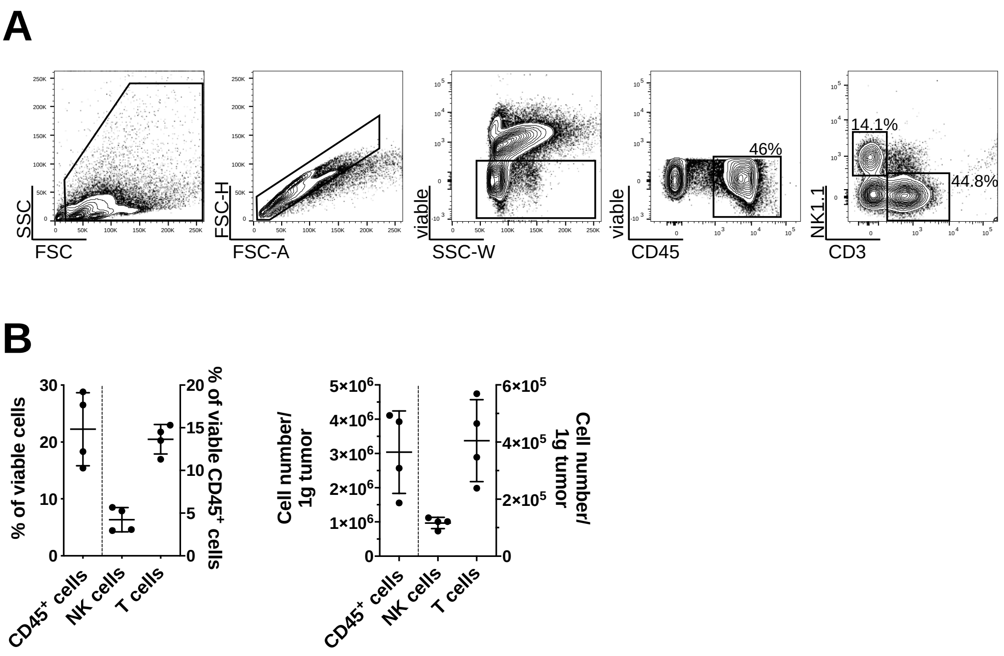

Supplementary Figure 1: (A) Gating strategy for the immune cell infiltrate. Immune cells are pre-gated on single, viable cells, then CD45 for all immune cells, followed by NK (NK1.1$^+$) as well as (CD3$^+$) T cells. Representative example for a BRAFi-sensitive tumor is shown. **(B)** Tumor-infiltrating CD45$^+$ cells, NK and T cells from tumors analyzed on day 8 after tumor transplantation, the time point when BRAFi treatment was initiated. Results are displayed as percentage as well as cell numbers/gram tumor. Summary graphs for one experiment are shown (n = 4).

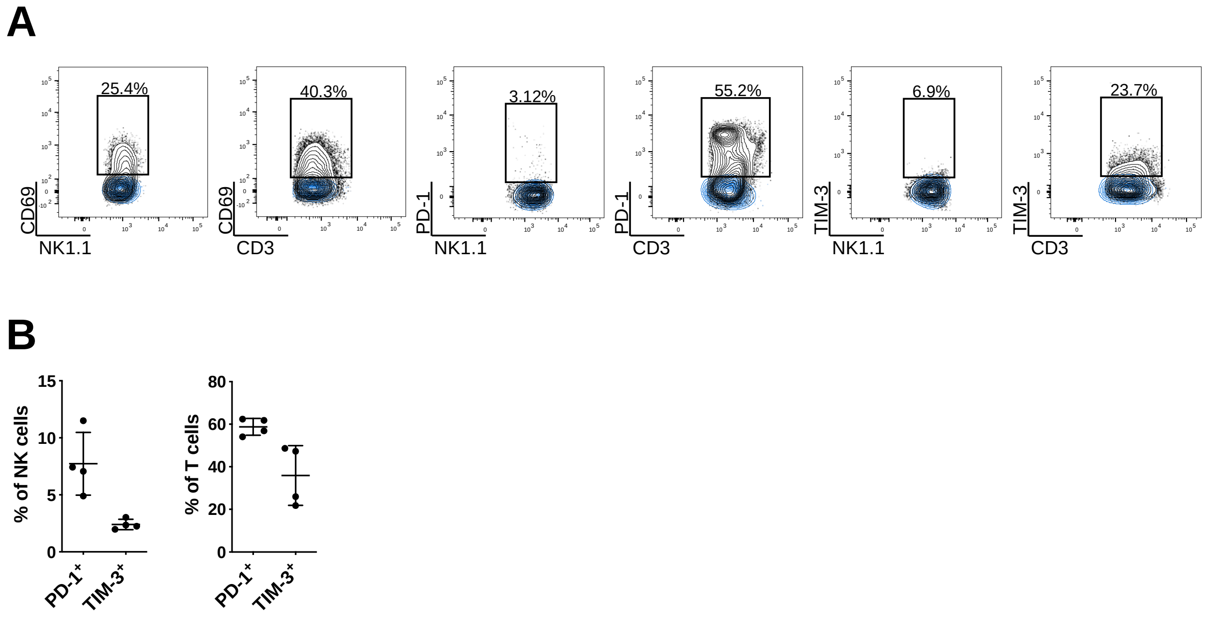

Supplementary Figure 2: (A) Gating strategy for CD69, PD-1 and TIM-3 is shown. Cells were pre-gated on single, viable CD45^{+} cells. Representative example for a BRAFi-sensitive tumor is shown. FMO or isotype-matched antibodies served as controls (blue). **(B)** Percentages of PD-1^{+} and TIM-3^{+} cells of NK and T cells infiltrating tumors dissected on day 8 after tumor transplantation were determined by flow cytometry analysis. Summary graphs for one experiment are shown (n = 4).

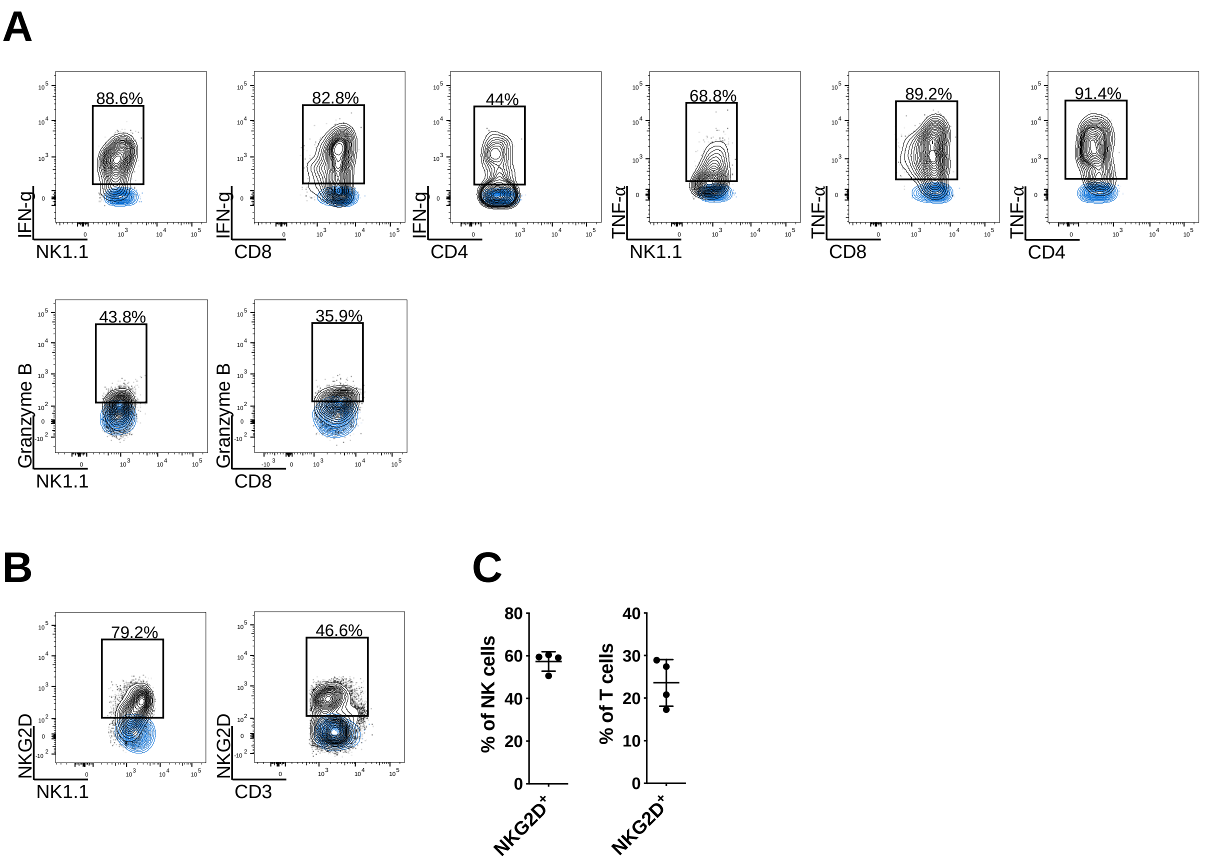

Supplementary Figure 3: (A and B) Gating strategy for (A) IFN-γ, TNF-α and granzyme B and (B) NKG2D is shown. Cells were pre-gated on single, viable $CD45^{+}$ cells. Representative example for a BRAFi-sensitive tumor is shown. FMO or isotype-matched antibodies served as controls (blue). **(C)** Percentages of $NKG2D^{+}$ cells of NK and T cells infiltrating tumors analyzed on day 8 after tumor transplantation were determined by flow cytometry analysis. Summary graphs for one experiment are shown (n = 4).

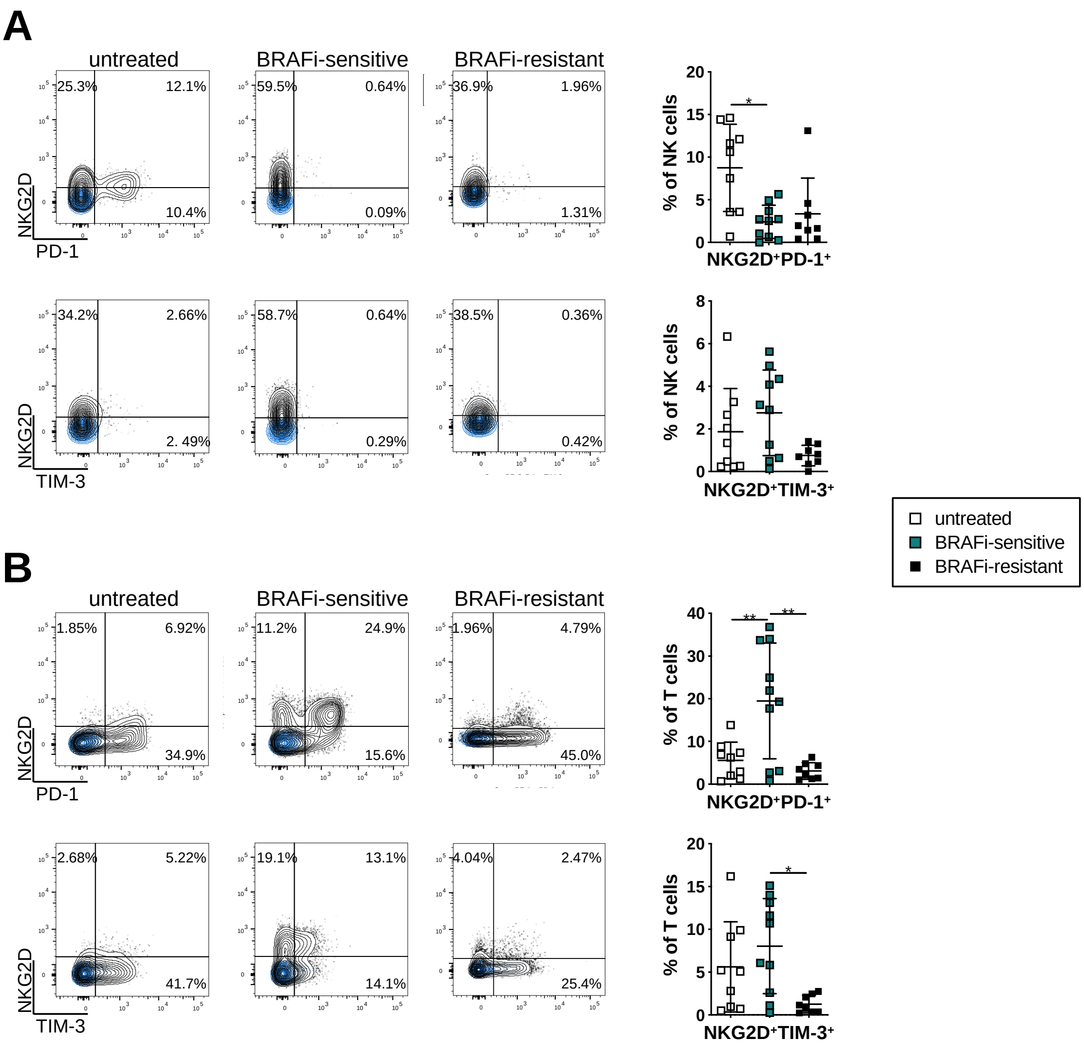

Supplementary Figure 4: (A and B) Gating strategy to determine co-expression of NKG2D with PD-1 or NKG2D with TIM-3. Percentages of double-positive cells of (A) NK cells and (B) T cells are shown. Dot plots show representative examples for an untreated, BRAFi-sensitive and BRAFi-resistant tumor. Cells were pre-gated on single, viable CD45$^+$ cells. FMO or isotype-matched antibodies served as controls (blue). Summary graphs for at least 2 independent experiments are shown (n ≥ 8/group).

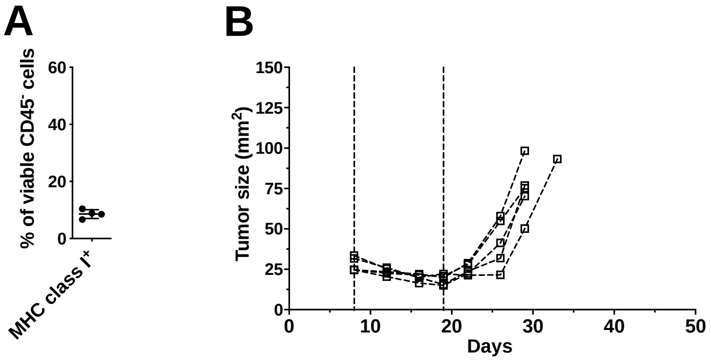

Supplementary Figure 5: (A) Percentages of MHC-class I$^+$ cells of viable CD45$^-$ tumor cells from tumors analyzed on day 8 after tumor transplantation were determined by flow cytometry analysis. Summary graph for one experiment is shown (n = 4). **(B)** Tumor growth curve upon s.c. injection with 3x10^5 murine melanoma D4M cells. On day 8 (dotted vertical line), mice were switched to BRAFi-containing chow and on day 19 (second dotted vertical line) mice were switched back to control chow (no BRAFi) and given s.c. peritumorally 2 mg/kg imiquimod (TLR7A). Summary graph for one experiment is shown (n = 5).
